# Fluorescence Immunoassay of Prostate-Specific Antigen Using 3D Paddle Screw-Type Devices and Their Rotating System

**DOI:** 10.3390/bios14100494

**Published:** 2024-10-11

**Authors:** Su Bin Han, Han Sol Kim, Young Ju Jo, Soo Suk Lee

**Affiliations:** Department of Pharmaceutical Engineering, Soonchunhyang University, Asan 31538, Republic of Korea; ant0921@sch.ac.kr (S.B.H.); itig2000@sch.ac.kr (H.S.K.); yjcho0214@sch.ac.kr (Y.J.J.)

**Keywords:** immunoassay, prostate-specific antigen, 3D paddle screw, fluorescence measurement, detection sensitivity

## Abstract

In this paper, we present a sensitive and highly reproducible fluorescence immunosensor for detecting PSA in human serum. A unique feature of this study is that it uses creatively designed paddle screw-type devices and their custom-made rotating system for PSA immunoassay. The paddle screw devices were designed to maximize the surface-to-volume ratio over which the immunoassay reaction could occur to improve detection sensitivity. This paddle screw-based immunoassay offers an accessible and efficient method with a short analysis time of less than 30 min. Active rotation of the paddle screw plays a crucial role in fast and accurate analysis of PSA. Additionally, a paddle screw-based immunoassay and subsequent fluorescence detection using a custom prototype fluorescence detection system were compared to a typical well plate-based immunoassay system. Results of PSA detection in human serum showed that the detection sensitivity through the paddle screw-based analysis improved about five times compared to that with a well plate-based analysis.

## 1. Introduction

Immunoassay is a type of biochemical test that uses a specific antibody–antigen binding reaction to detect the presence of specific molecules (antigens) in a sample [1,2,3]. Although many different types and formats of immunoassays have been reported, they are all based on the basic principle of antibody-–antigen specificity. Among them, the sandwich immunoassay is the most commonly used method for detecting macromolecular antigens. It is very important to develop an immunoassay that can efficiently detect biomolecules with high sensitivity and high reproducibility. In particular, in terms of early diagnosis of disease, the development of a new approach that can improve detection sensitivity so that low concentrations of disease-related biomolecules can be detected using an immunoassay is one of the main goals of researchers [4,5]. Researchers have developed a variety of successful signal amplification strategies, with enzyme- and fluorophore-labeling methods being the two most prominent ones [6,7,8]. Enzyme-linked immunosorbent assay (ELISA) is one of the most widely used approaches for detecting biomolecules [9,10,11,12]. It is excellent for situations in which multiple samples need to be tested at the same time. However, this format involves a series of steps, including sample introduction, incubation, and washing, which can be time-consuming. In addition, it sometimes suffers from low sensitivity [13]. To overcome these drawbacks, various efforts have been made to develop new immunoassay methods, with fluorescence immunoassay being one of them.

Fluorescence immunoassay (FIA) is also widely used in the diagnosis of endocrine and metabolic diseases due to its advantages such as high specificity for immune response and the high sensitivity of the fluorescence technology. Fluorophores such as quantum dots and organic fluorescent dyes can be coupled to detect antibodies in a sandwich fluorescence immunoassay format, as fluorescent signals can be emitted by appropriate light irradiation [14]. Among various efforts to achieve higher biomolecule detection sensitivity, a high surface-to-volume ratio, which allows for a higher surface area for specific biomolecule immobilization, is of particular interest, as it can increase the sensitivity of a fluorescence immunoassay. For this purpose, methods using magnetic nanoparticles, precious metal nanoparticles, and microbeads have been developed [15].

**Scheme 1:**
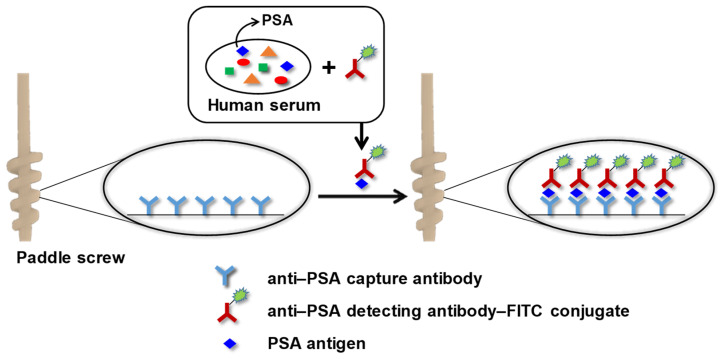
Schematic illustration of a sandwich immunoassay reaction for PSA occurring on the surface of a paddle screw-type device utilized in this study.

Here, we demonstrate a dramatic improvement in the speed and sensitivity of a fluorescence immunoassay for detecting prostate-specific antigen (PSA) using a uniquely designed paddle screw-type device and a rotation system. PSA is a blood protein produced by normal and malignant cells of the prostate. It is most widely used as an initial screening tool to detect prostate cancer and benign prostatic hyperplasia through routine blood analysis, with a recommended cutoff value of 4 ng/ mL [16]. A schematic diagram of the sandwich immunoassay reaction for detecting PSA occurring on the surface of a paddle screw-type device is presented in Scheme 1. The paddle screw surface undergoes immobilization of anti-PSA capture antibodies, binding of target PSA at various concentrations, and reacting with FITC-conjugated detection antibodies. Additionally, quantitative detection of PSA was performed using a customized fluorescence detection system.

## 2. Materials and Methods

### 2.1. Reagents and Materials

For the PSA immunoassay, a recombinant human PSA protein (ab283430), an anti-PSA monoclonal capture antibody (ab242191) and an anti-PSA monoclonal detecting antibody (ab242192) were purchased from Abcam (Cambridge, UK). PSA-free human serum for PSA spiking at each concentration was obtained from BioChemed Services (Winchester, VA, USA). Bovine serum albumin (BSA), 3-glydoxypropyltriethoxysilane (3-GPTES), 1,4-dithiothreitol (DTT), and fluorescein isothiocyanate (FITC) were purchased from Sigma-Aldrich (St. Louis, MO, USA). PBS buffer was obtained from Thermo Fisher Scientific (Waltham, MA, USA). Organic solvents such as methanol and absolute ethanol were purchased from Samchun Pure Chemical Co. Ltd. (Seoul, Republic of Korea). All aqueous solutions for PSA immunoassay were prepared with double-distilled water from a Milli-Q water-purifying system (18 MΩ cm, Millipore Corp., Bedford, MA, USA).

### 2.2. Preparation of Antibodies-Conjugated Paddle Screw

**Figure 1:**
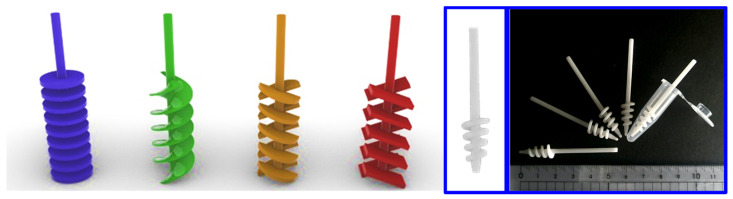
Various shapes of 3D paddle structures. The paddle screw shown in the blue box was selected considering that it was convenient to manufacture it with 3D printing and that it matched well with 1.5 mL microtubes for PSA immunoassay.

Paddle screws made of acrylonitrile butadiene styrene (ABS) were manufactured using a 3D printing method. Among various shapes of 3D paddle structures, the paddle screw shown in the blue box of Figure 1 was selected because it was convenient to manufacture it by 3D printing. In addition, it matched well with a 1.5 mL microcentrifuge tube. These 3D printed paddle crews were thoroughly washed by sonication in ethanol and double-distilled water (each 3 times × 5 min at room temperature) to remove any debris generated during fabrication, dried under nitrogen, and stored in a desiccator until use. These cleaned paddle screws were activated in a UV ozone cleaner (144AX-220; Jelight Company, Inc., Irvine, CA, USA) for 10 min. Activated paddle screws were allowed to stand in a solution of freshly prepared 3% (vol./vol.) 3-GPTES in ethanol for 1 h, followed by rinsing with ethanol for 2 min and drying under nitrogen. These silanized paddle screws were oven-baked at 110 °C for 1 h, followed by rinsing with ethanol for 2 min and drying under nitrogen. For immobilization of the captured antibody on the surface of paddle screws, 3-GPTES modified paddle screws were soaked in a solution of anti-PSA antibody (ab242191, 100 µg/mL in phosphate-buffered saline (PBS), pH 8.0) and incubated at 4 °C overnight or in a humidity chamber at room temperature for one hour. After incubation, paddle screws were rinsed with PBS and deionized water to remove physically absorbed antibodies. Sequentially, empty sites where antibodies were not immobilized within the sensing area were blocked with 3% BSA in a PBS solution (pH 8.0) at room temperature for one hour with shaking to prevent non-specific binding of proteins in subsequent steps.

### 2.3. Fluorescence Measurement System

**Figure 2:**
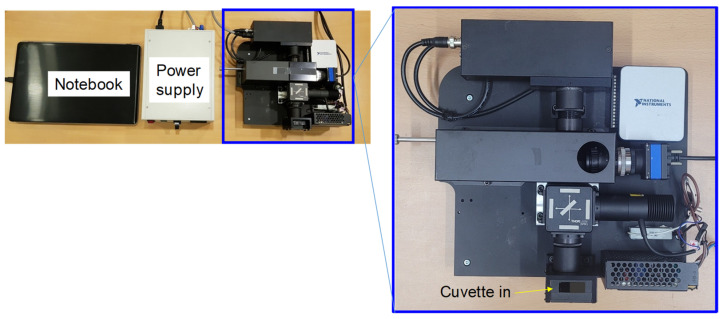
A custom-made fluorescence detection system consisting of an LED light source, fluorescence filters, and a photodiode detector.

Fluorescence intensity was continuously measured with a custom-made optical system. The incident light from an LED (M470L3-470 nm, 650 mW (Min) Mounted LED, 1000 mA, Thorlabs, Newton, NJ, USA) with a short pass filter (DFM1-Kinematic Fluorescence Filter Cube for Ø25 mm Fluorescence Filters, 30 mm Cage Compatible, Right-Turning, 1/4″-20 Tapped Holes, Thorlabs) provided excitation. Emitted light was measured with a photodiode (DET10A-Si Detector, 200–1100 nm, 1 ns Rise Time, 0.8 mm^2^, 8–32 Taps, Thorlabs) through a long pass filter (Figure 2). The wavelength of excitation light for FITC was 488 nm and that of emission fluorescence was 514 nm. We compared our fluorescence measurement system with a conventional Horiba FluoroMax-4 spectrofluorometer (HORIBA Scientific, Edison, NJ, USA). An FLx800 fluorescence microplate reader (BioTek, Winooski, VT, USA) was also used. The results were compared with those of well plate-based fluorescence ELISA.

### 2.4. FITC Conjugation of Anti-PSA Detecting Antibody

Anti-PSA detecting antibody was conjugated with fluorescein isothiocyanate (FITC) according to a previously described method [17] with minor modifications. Briefly, 1 mg of anti-PSA antibody (ab242192) in 1.0 mL of 500 mM carbonate buffer (pH 9.5) was mixed with 200 μL of FITC in freshly prepared DMSO. The reaction tube was wrapped in foil and incubated in a shaker at room temperature for 90 min. The mixture was then passed through a Sephadex G25 column to remove unreacted FITC and collect FITC–anti-PSA antibody conjugate in the void volume. Finally, the FITC–anti-PSA antibody conjugate was exchanged into storage buffer (PBS, pH 7.4).

### 2.5. Immunoassay for Detecting PSA Using a Paddle Screw Device and Its Rotating System

**Figure 3:**
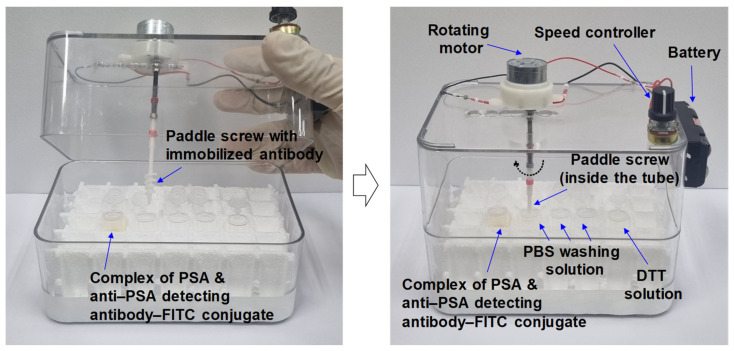
A custom-made paddle screw rotating system. Microtubes (1.5 mL) for PSA assay located at the bottom of the system consisted of one solution containing complex of PSA and anti-PSA detecting antibody–FITC conjugates, three PBS buffer solutions, and one DTT solution.

For detecting PSA in human serum, an anti-PSA capture antibody immobilized paddle screw and a custom-made rotating system were prepared. As shown in Scheme 1, 500 μL of PSA-free human serum containing PSA was mixed with 500 μL of FITC conjugated anti-PSA detecting antibody in a 1.5 mL microcentrifuge tube for 5 min, allowing the PSA to bind to its specific detecting antibody–FITC conjugate. The anti-PSA capture antibody-immobilized paddle screw was first immersed in a tube containing a complex formed from PSA and its specific detection antibody–FITC conjugate. The paddle screw was then rotated at 200 rpm for 10 min to allow the detection antibody–PSA complex to bind to the immobilized anti-PSA capture antibody and form a sandwich format immune complex. The paddle screw was moved into a PBS solution and washed for 1 min while rotating at 1000 rpm. The same process was repeated three times. After the washing process, the paddle screw was moved again into a 50 mM DTT solution in deionized water and rotated at 200 rpm for 10 min to break the disulfide bond, thereby releasing the FITC moiety from the paddle screw. All of the above processes using paddle screws were accomplished through a custom-made rotating system as shown in Figure 3, which consisted of a rotating motor, a speed controller, and a battery.

Experiments for each PSA concentration (1.0 pg/mL to 25 ng/mL) were performed in triplicate over different days. We also performed blind spiked sample tests using a combination of a paddle screw-type device with a custom fluorescence measurement system and a combination of conventional well plates with a FLx800 fluorescence microplate reader (BioTek, Winooski, VT, USA). A blind test was conducted using a combination of the two immunoassay systems mentioned above for five samples prepared by adding unknown amounts of PSA to PSA-free human serum at different concentrations.

### 2.6. Fluorescence ELISAs for Detecting PSA Using Well Plates

Fluorescence ELISAs for detecting PSA were carried out using Immulon 2HB 96-well microtiter plates (Thermo Fisher Scientific, Waltham, MA, USA). In these studies, ELISA plates were coated with 100 μL of anti-PSA capture antibody in pH 7.4 phosphate-buffered saline (PBS) overnight at 4 °C. Each well was washed three times with pH 7.4 PBS buffer and blocked with 5% bovine serum albumin (BSA) in PBS for 1 h at room temperature (RT). Finally, the plates were washed twice with PBS buffer. After human serum samples (50 μL) spiked with varying concentrations of PSA were added to appropriate wells, the plates were incubated at RT for one hour. After incubation, the plates were washed three times with PBS, added with 50 μL of anti-PSA detecting antibody conjugated with FITC, and incubated at RT for one hour. Each well was washed three times with PBS. Fluorescent signals were read at 514 nm with an FLx800 fluorescence microplate reader.

## 3. Results and Discussion

### 3.1. PSA Immunoassay Using Paddle Crews and a Rotating System

**Figure 4:**
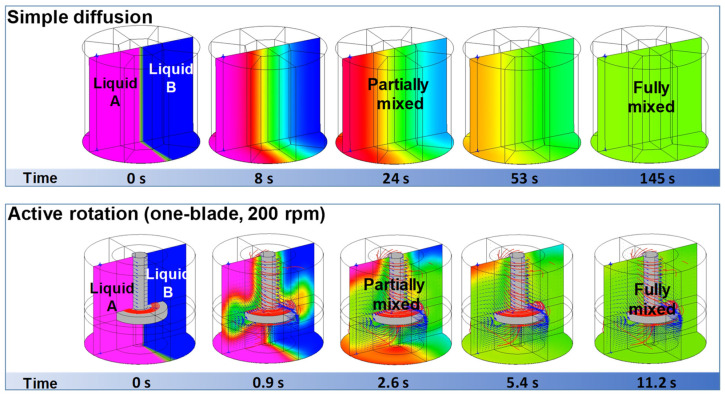
Computational fluid dynamics (CFD) simulations for mixing effects of two liquids. The difference in mixing effect between the two liquids due to simple diffusion and active rotation with a single-blade paddle (200 rpm) was approximately 13 times.

Scheme 1 shows analysis of PSA based on the sandwich immunoassay format using an anti-PSA capture antibody and a FITC-conjugated anti-PSA detecting antibody on the surface of paddle screws with fluorescent detection of released fluorophore from paddle screws after treatment with DTT. Sensitive detection of PSA could be achieved based on this classical sandwich immunoassay and subsequent fluorescent detection. All of the above immunoassay and DTT treatment processes were accomplished using paddle screws and a custom-made rotating system, as shown in Figure 3. The paddle screw-based immunoassay offers an accessible and efficient method with an analysis time of less than 30 min. Active rotation of the paddle screw plays a crucial role in fast analysis. For example, the difference in mixing effect between two liquids resulting from simple diffusion and active rotation (200 rpm) based on computational fluid dynamics (CFD) simulations was about 13 times (Figure 4).

### 3.2. Dependence of Fluorescence Response on PSA Concentration

**Figure 5:**
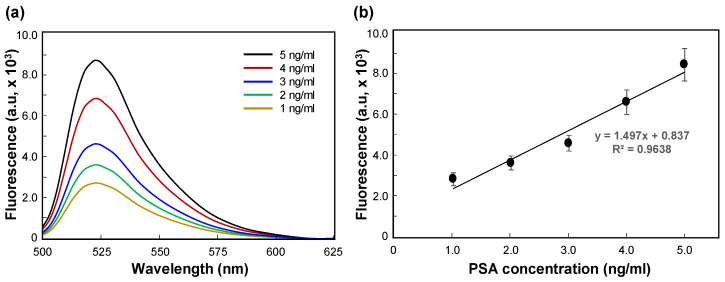
(**a**) Fluorescence spectra of FITC released by DTT treatment after sandwich immunoassay according to PSA concentration, and (**b**) fluorescence intensity versus PSA concentration.

Figure 5a shows the results of fluorescence spectra of FITC released by DTT treatment after sandwich immunoassay according to PSA concentration. As the PSA concentration increased from 1 ng/mL to 5 ng/mL, the fluorescence emission peak intensity also increased. The results revealed that higher concentrations of PSA promoted the capture of more FITC fluorophores, thereby emitting higher fluorescence. Fluorescence intensity versus PSA concentration is shown in Figure 5b. Fluorescence intensity at 520 nm and PSA concentration showed a linear relationship with a constant coefficient of 0.9638.

**Figure 6:**
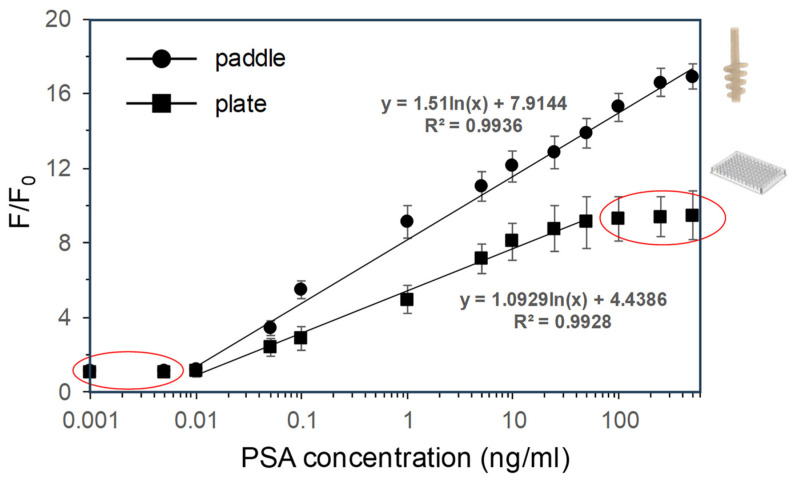
Changes in fluorescence signal ratio in the paddle screw-based immunoassay according to PSA concentration. The lower plot (solid square marks) shows the results of a well plate sandwich immunoassay. The upper plot (solid circle marks) shows the results of a paddle screw-based sandwich immunoassay. Data in red circles deviated from the logarithmic linear range.

The effects of different concentrations of PSA ranging from 1.0 pg/mL to 500 ng/mL (1.0 pg/mL, 5.0 pg/mL, 10 pg/mL, 50 pg/mL, 100 pg/mL, 1.0 ng/mL, 5 ng/mL, 10 ng/mL, 25 ng/mL, 50 ng/mL, 100 ng/mL, 250 ng/mL and 500 ng/mL) spiked in PSA-free human serum on changes in fluorescence signal ratio (*f/f*_0_, *f* = average fluorescence intensity at the tested PSA concentration; *f*_0_ = fluorescence intensity of the blank sample) in the sandwich immunoassay were also investigated. As a blank, the entire process was performed with a serum without PSA spiking. We used average values obtained from results of three repeated experiments in all applied concentrations of PSA. In this assay, the FITC fluorophore had a peak emission fluorescence at 520 nm. The solid circle marks in Figure 6 are the results of a PSA immunoassay conducted with a paddle screw device and solid square marks are results of a well plate-based analysis. In each case, the same process and reaction time were applied, and the ratio of fluorescence signals was analyzed with our custom-made fluorescence measurement system. As the PSA concentration increased from 10.0 pg/mL to 500 ng/mL, the change in fluorescence signal ratio also increased logarithmically in paddle screw devices. The higher the concentration of the target PSA, the more PSA was captured by capture antibodies. Eventually, as the concentration of PSA increased, the amount of fluorophore released from the surface of paddle screws or well plates after the completion of the sandwich immunoassay with subsequent DTT treatment also increased. However, in the well plate, the change in fluorescence signal ratio increased logarithmically as the PSA concentration increased from 10.0 pg/mL to only 50 ng/mL. These results indicate that the immobilization density of the capture antibody to paddle is relatively higher than that of the plate due to a significant increase in the surface-to-volume ratio of the paddle structure. The limit of detection (LOD) for PSA immunoassay in human serum was 13.7 pg/mL for a paddle screw assay and 68.6 pg/mL for well plate assay, respectively. Here, the LOD was calculated according to Shrivastava and Gupta [18] with Equation (1):

LOD = MEAN_blank_ + 1.645(SD_blank_) + 1.645(SD_low concentration sample_) (1)

The low concentration sample is the lowest concentration in the logarithmic linear range, representing a PSA concentration of 5.0 pg/mL in human serum. The paddle screw-based PSA immunoassay had an LOD that was approximately 5.0 times lower, which was believed to be due to a difference in standard deviation of a low concentration of the PSA sample. The higher sensitivity of the paddle screw-based PSA immunoassay was due to a high surface-to-volume ratio and increases in the number of antibody and PSA molecules participating in the immune reaction per given reaction time due to rotation of the paddle. Reproducibility of repeated experiments at each PSA concentration was expressed as coefficient of variation (CV). The results are summarized in the Supplementary Materials (Table S1). The %CV values for each PSA concentration were lower in paddle tests than in plate-based tests. Because the fluorescent signal intensities of the paddle, which has a high surface-to-volume ratio, are relatively large compared to those of the plate, the error value of each experiment was relatively small. These results revealed that the paddle screw-based PSA biosensors developed in this study had an LOD similar to or better than several types of PSA biosensors reported recently [19,20,21,22,23,24,25], as shown in Table 1.

### 3.3. Blind Sample Analysis and Recovery Test for PSA Spiked in Human Serum

**Figure 7:**
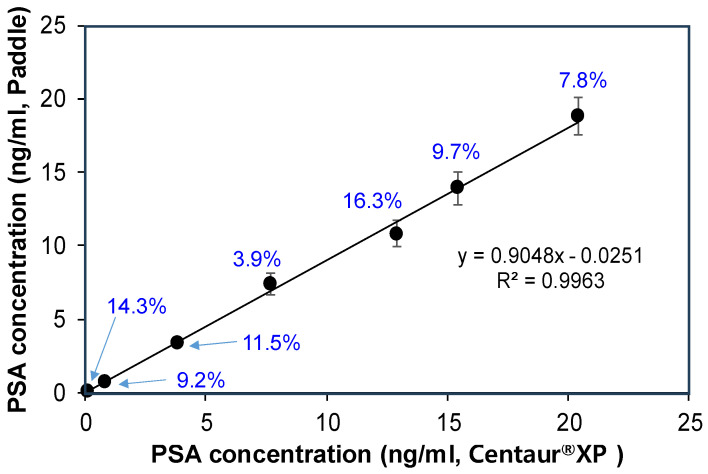
Correlation of the proprietary paddle screw system with the commercial Centaur^®^XP immunoassay system in PSA immunoassays for seven blinded samples prepared at different concentrations. A reasonable correlation was observed between the two methods over the entire tested concentration range. The percentage above the solid circle represents the relative error at each concentration.

To verify the accessibility and accuracy of the paddle screw-based PSA immunoassay, we carried out a blind spiked sample test using our paddle screw and rotating system for immunoassay and a custom-made fluorescence measurement system. In addition, the Centaur^®^XP immunoassay system (Siemens Diagnostics, Tarrytown, NY, USA), used in clinical practice, was used as a validation experiment to confirm our results. Five samples were prepared by spiking an unknown amount of PSA into PSA-free human serum and tested independently using the two separate systems mentioned above (paddle screw and Centaur^®^XP). Changes in fluorescence intensity ratios resulting from testing seven samples in the paddle screw-based immunoassay and custom-made fluorescence measurement system were converted to corresponding concentrations of PSA using the solid circle marks shown in Figure 6 as a standard curve. The concentrations obtained from two separate immunoassays and corresponding measurement systems are summarized in Figure 7 and Table 2. Here, the average value of three repeated measurements in the paddle screw immunoassay system was used. The relative error was calculated by dividing the difference between values obtained from the two methods based on measurements made using the Centaur^®^XP system and multiplying the result by 100.

The results obtained with the paddle screw system were slightly lower than those measured by Centaur^®^XP commonly used in clinical practice. The relative error was as high as 10% to 20%. Despite these high relative errors, acceptable agreement was observed between the two immunoassay systems over the entire tested range of PSA concentrations, as shown in Figure 7. In Deming regression analysis, measured PSA concentrations from the two different immunoassay systems showed a significant correlation, with a correlation coefficient of 0.9963 and a slope of 0.9048. Considering differences in these two immunoassay procedures and measurement systems, a proportional bias of 0.9048 observed in the regression analysis was fully acceptable [26,27,28]. Finally, recovery rates of five concentrations (25 pg/mL, 50 pg/mL, 100 pg/mL, 250 pg/mL, and 500 pg/mL) of PSA spiked into PSA-free serum in the paddle screw immunoassay system ranged from 90.8% to 93.4% (Table 3).

## 4. Conclusions

In this study, we successfully implemented a unique paddle screw-based sandwich immunoassay and fluorescence detection using a custom-made fluorescence detection system for highly sensitive analysis of PSA in human serum. The use of specially designed paddle screws and a rotating system significantly decreased the reaction time of the immunoassay process. An increase in detection sensitivity was caused by an increase in the surface-to-volume ratio. The LOD of the PSA immunoassay in the human serum was 13.7 pg/mL using the paddle screw system and 68.6 pg/mL in the well plate system. Due to its high sensitivity and fast assay process, this paddle screw-based sensing system is expected to be a useful platform for most immunoassays for detecting various biomarkers, with practical advantages of economic impact, such as a low-cost paddle fabrication, a short analysis time due to active rotation of the paddle, and an ability to be reused by plasma treatment after analysis is completed. However, the efficiency of large-scale screening is still lacking. Therefore, our future research needs to focus on improving the performance of the paddle screw-based biosensor system, with an ability to detect multiple targets simultaneously, and miniaturizing the paddle screw device to enable point-of-care testing. Excluding the DTT processing step will shorten the overall analysis time. To achieve this, it is essential to develop an analysis container made of transparent material to enable direct detection of fluorescence on the paddle screw.

## Figures and Tables

**Table 1:** Comparison of LODs for PSA detection with reported methods.

Detection Technique	LOD (pg/mL)	Dynamic Range (ng/mL)	Reference
Absorbance	202	1–40	[19]
Electrochemistry	38	0.1–100	[20]
Electrochemistry	150	0.01–20	[21]
Fluorescence	4.4	0.05–8.0	[22]
Fluorescence	5.6	0–25	[23]
QCM ^1^	48	0.1–25	[24]
SPR ^2^	9	0.01–20	[25]
ECL ^3^	0.1	0.001–100	[26]
Fluorescence	13.7	0.01–500	This work

^1^ QCM: Quartz crystal microbalance; ^2^ SPR: surface plasmon resonance; ^3^ ECL: electrochemiluminescence.

**Table 2:** Blind-spiked PSA sample tests using our paddle screw system and the Centaur^®^XP immunoassay system.

Sample No.	Centaur^®^XP (ng/mL)	Paddle Screw (ng/mL)	Relative Error (%) ^1^
1	0.0914	0.0783 ± 0.00487	14.3
2	0.841	0.764 ± 0.0594	9.2
3	3.85	3.41 ± 0.298	11.5
4	7.69	7.39 ± 0.715	3.9
5	12.9	10.8 ± 0.894	16.3
6	15.4	13.9 ± 1.095	9.7
7	20.4	18.8 ± 1.248	7.8

^1^ Relative error: (Centaur^®^XP − Paddle screw)/Centaur^®^XP × 100.

**Table 3:** Recovery test for accuracy of the paddle screw immunoassay system for PSA detection.

Sample No.	Added (pg/mL)	Found (pg/mL)	Recovery Rate (%) ^1^
1	25	22.7 ± 0.33	90.8
2	50	45.9 ± 0.62	91.8
3	100	93.1 ± 0.96	93.1
4	250	230.6 ± 3.28	92.2
5	500	466.9 ± 5.42	93.4

^1^ Recovery rate: (found/added) × 100.

## Data Availability

Data are contained within the article.

## Supplementary Materials

The following supporting information can be downloaded at https://www.mdpi.com/article/10.3390/bios14100494/s1, Table S1: Data for PSA immunoassay using the paddle screw-type devices and well-plates.
