# Supplementary material for: Fluorescence Immunoassay of Prostate-Specific Antigen Using 3D Paddle Screw-Type Devices and Their Rotating System
Source: Biosensors (Basel). 2024 Oct 11;14(10):494. doi: 10.3390/bios14100494 (PMC11506760; doi:10.3390/bios14100494)
Supplement: Supplementary file 1 [file biosensors-14-00494-s001.zip › biosensors-3189637-supplementary.pdf]

biosensors

MDPI

Article

Fluorescence Immunoassay of Prostate-Specific Antigen Using 3D Paddle Screw-Type Devices and Their Rotating System

Su Bin Han, Han Sol Kim, Young Ju Jo and Soo Suk Lee *

Department of Pharmaceutical Engineering, Soonchunhyang University, Asan 31538, Republic of Korea; ant0921@sch.ac.kr (S.B.H.); itig2000@sch.ac.kr (H.S.K.); yjcho0214@sch.ac.kr (Y.J.J.)

* Correspondence: sslee0810@sch.ac.kr; Tel.: +82-41-530-1394

Table S1. Data for PSA immunoassay using the paddle screw-type devices and well-plates.

PSA Conc. (ng/ml)	F/F0 (Paddle)	Std.	F/F0 (Plate)	Std.
0 (blanck)	1	0	1	0
0.001	1.12	0.1	1.06	0.24
0.005	1.17	0.15	1.09	0.19
0.01	1.24	0.15	1.16	0.31
0.05	3.41	0.38	2.41	0.49
0.1	5.47	0.46	2.87	0.62
1	9.13	0.88	4.96	0.78
5	11.03	0.81	7.14	0.76
10	12.12	0.83	8.08	0.99
25	12.83	0.87	8.76	1.24
50	13.87	0.82	9.1	1.37
100	15.27	0.78	9.28	1.2
250	16.58	0.76	9.39	1.08
500	16.91	0.68	9.46	1.32
